# Dissociative experiences mediate the association between childhood trauma and verbal hallucinations, but not delusional thoughts, in borderline personality disorder

**DOI:** 10.3389/fpsyt.2025.1532234

**Published:** 2025-08-26

**Authors:** Katrin Schroeder, Anja Schätzle, Ingo Schäfer, Christian G. Huber

**Affiliations:** ^1^ Department of Psychiatry and Psychotherapy, University Medical Center Hamburg-Eppendorf, Hamburg, Germany; ^2^ Universitäre Psychiatrische Kliniken Basel, University of Basel, Basel, Switzerland

**Keywords:** dissociation, auditory verbal hallucination (AVH), delusions, adverse childhood events, borderline personality disorder

## Abstract

**Introduction:**

Auditory verbal hallucinations (AVH), a disturbance of auditory perception, and delusions, a content-related thought disorder, are common in borderline personality disorder (BPD). However, they are not as thoroughly studied and clinically acknowledged as other symptoms. Associations between childhood trauma, dissociative symptoms, and AVH—as well as delusions—have been reported in schizophrenia but remain understudied in BPD.

**Methods:**

We calculated Pearson’s correlations and tested the mediating effects of dissociative symptoms, assessed with the Dissociative Experiences Scale (DES), on the association between childhood trauma, assessed with the Childhood Trauma Questionnaire (CTQ), and both AVH and delusions. A total of 74 BPD patients were examined using the Psychotic Symptoms Rating Scale (PSYRATS) interview. For the mediation analyses, Preacher and Hayes’ SPSS bootstrap macro was used to estimate the significance of the mediator.

**Results:**

AVH were reported by 10 patients (13.5%) and delusional thoughts by eight patients (10.8%). In the mediator analyses, dissociative experiences significantly mediated the association between childhood trauma and auditory verbal hallucinations, with an unstandardized regression coefficient between CTQ-Total and DES-Total of *b* = 10.0083; *p* = 0.0008 and between DES-Total and PSYRATS-AVH of *b* = 0.0102; *p* = 0.0009. The relationship between CTQ-Total and the PSYRATS-Delusions Scale was not significantly mediated by DES-Total.

**Discussion:**

The results of the mediation analyses are similar to those shown in schizophrenia, suggesting that the examined symptoms in BPD may underlie similar mechanisms. Further research should examine the benefit of the therapeutic approaches for each of the trauma-associated symptom clusters.

## Introduction

Auditory verbal hallucinations (AVH) are defined as the perception of spoken words without an external stimulus in a fully conscious state (1). They are a core symptom of schizophrenia but can also occur in various other psychiatric illnesses, e.g., such as bipolar disorder, major depressive disorder, substance use disorders, dissociative disorders, and dementia (2–4). Delusions are a content-related thought disorder where reality testing is impaired. They are defined as having “a false firm conviction […] whose truth is immediately evident to the person and requires no justification […, while the] environment does not share the belief and in the cultural context, the opinion is considered false” (5). Delusions are often considered prototypical psychotic phenomena and can occur in a range of mental illnesses, such as schizophrenia spectrum disorders, affective disorders, substance use disorders, and dementia, but are not typically observed in dissociative disorders (5). Both AVH and delusions are common symptoms in borderline personality disorders (BPD). Approximately 30% of BPD patients experience delusions, and about 50% experience hallucinations (6). However, most studies were conducted in small samples (7) or focused only on AVH (8). AVH in BPD has been found to burden affected patients to a degree comparable to that experienced by patients with schizophrenia (8–10). Additionally, it was found that the phenomenology of the AVH in BPD—e.g., conviction, frequency, or beliefs about the location of the auditory hallucination (8, 9, 11–13), or whether it was a known or unknown person (14)—was comparable to AVH in schizophrenia.

While both the Diagnostic and Statistical Manual of Mental Disorders, Fourth and Fifth Edition (DSM-IV and DSM-5) criteria (15, 16) describe AVH and delusions as transient in BPD, studies exploring the duration of these symptoms show different results (8, 10–13, 17–21). The symptoms examined lasted at least several weeks (18) and up to 18 years (8). Although the burden caused by these symptoms in BPD patients has been examined only with regard to AVH so far, it is clear that these experiences have a significant impact (8, 9, 11, 21, 22). For instance, they have been shown to be associated with suicidal behavior, a key problem in the treatment of borderline patients (10, 23–25).

Another cluster of symptoms commonly found in BPD is dissociative symptoms [e.g., (10, 26–30)]. Associations have been found between these symptoms and childhood sexual abuse (31–33), as well as other forms of childhood maltreatment (30, 34, 35). Dissociation is considered an important element for understanding the relationship between trauma and hallucinations, as well as delusions. However, the relationship between trauma, dissociation, and these symptoms has primarily been studied in schizophrenia: the interrelation of the clinical characteristics, dissociation, and childhood trauma has been the subject of several investigations in schizophrenia [e.g., (24, 36, 37)]. It has been discussed that severe dissociation may produce hallucinations or delusions, or could be a mediating factor in their development [e.g., (38)]. Allen et al. (39) emphasized the special role of dissociative detachment in this context. They suggested that dissociative detachment “undermines the individual’s grounding in the outer world, thereby hampering reality-testing and rendering the individual with posttraumatic symptoms vulnerable to the nightmarish inner world” [(39), p. 332)]. Empirical evidence for the relationship between dissociation and hallucinations/delusions comes, for instance, from a study by Perona-Garcelan and colleagues (40). In a mediation analysis, these authors found that dissociative symptoms were a mediator between childhood trauma and hallucinations, but not between childhood trauma and delusions, in a sample of patients with schizophrenia.

Although AVH, delusions, and dissociative symptoms are also prevalent in BPD, and although the majority of BPD patients have suffered some kind of childhood maltreatment (41–44), no studies exist, to our knowledge, that have examined potential associations between these factors in BPD. The aim of our study, therefore, was to examine whether dissociative symptoms have a mediating effect between childhood maltreatment and the development of AVH and delusions, respectively, in patients with BPD. We hypothesized that, given the similarities between AVH in schizophrenia and BPD, the associations between childhood maltreatment and the occurrence of dissociative and psychotic symptoms would be comparable to those in schizophrenia [e.g., (8, 9, 14)].

## Methods

### Participants

All participants were inpatients of a specialized ward for personality disorders at the University Medical Center Hamburg-Eppendorf, Germany. Inclusion criteria were a diagnosis of BPD according to the Diagnostic and Statistical Manual of Mental Disorders, Fourth Edition, Text Revision (DSM-IV-TR) (16), age between 18 and 65 years, and sufficient German language abilities. Exclusion criteria were organic mental disorders, acute intoxication, withdrawal syndrome, dementia, acute suicidality, and schizophrenia spectrum disorders. The final sample consisted of *n* = 74 patients.

All subjects provided written informed consent. The study was approved by the responsible ethics committee (State Chamber of Physicians, Hamburg, Germany).

### Procedures and assessments

The *diagnosis of BPD* according to the DSM-IV-TR (16) was confirmed using the German version of the Structured Clinical Interview for DSM-IV Axis I Disorders [SCID-l; (45)]. It comprises a screening questionnaire and a structured interview. The criteria are encoded as “1” (not met), “2” (partially met), or “3” (met). To diagnose a personality disorder, a minimum number of criteria must be met.

All patients were screened for *comorbid schizophrenia* sp*ectrum disorders* using the German version of Sections B and C of the SCID-I (45), a structured interview for Axis I disorders. Those who fulfilled the criteria for a schizophrenia spectrum disorder were excluded from the sample.

*Psychotic symptoms* were assessed using the Psychotic Symptom Rating Scales (PSYRATS). The PSYRATS is a semistructured interview consisting of 17 items, which are assigned to two subscales assessing AVH and delusion. AVH is evaluated with 11 items, and delusional beliefs with six items. Each item is evaluated on a 5-point scale. The PSYRATS has been evaluated as “a reliable and valid assessment tool for delusions and hallucinations” (46).

*Dissociative symptoms* were measured with the German version of the Dissociative Experiences Scale [DES; (47)]. This reliable and internally consistent self-report questionnaire is the most widely used instrument for dissociative symptoms in clinical samples. It contains items referring to amnesia, depersonalization, derealization, absorption, and identity alteration and comprises three subscales (absorption, depersonalization, and amnesia). The German version of the DES yields good to excellent statistical parameters, similar to the original version (48).

For assessing the type and severity of *childhood trauma*, the Childhood Trauma Questionnaire [CTQ; (49)] was used. This self-report questionnaire comprised 28 items assessing physical, sexual, and emotional abuse, as well as physical and emotional neglect. The items are rated on a 5-point scale (1 = never true to 5 = very often true). Strong psychometric properties have been demonstrated for the CTQ in both clinical and community samples (50). We categorized the CTQ scores for each subscale as proposed by the authors [“none or low”, “low to moderate”, “moderate to severe”, “severe to extreme”; (49)].

### Data analysis

Descriptive statistics are given in total numbers and percentages for nominal scaled variables, and in mean/median and standard deviation (SD) for ordinal and interval-scaled variables. In addition, SEM is reported where relevant.

In a first step, we examined correlations between childhood trauma, dissociation, AVH, and delusions using Pearson’s *r*. Effect sizes were defined as small (*r* < 0.3), medium (*r* = 0.3–0.5), and large (*r* ≥ 0.5), according to Cohen (51).

To test our main hypotheses, we used a mediation analysis. In the simple mediation model, the total DES score was the mediating variable, and childhood trauma was the independent variable. We used Hayes’s (52) SPSS bootstrap macro to estimate mediator significance with a 95% confidence interval (CI) and 10,000 bootstrap samples. According to the authors, if the 95% CI does not include zero, the effect is significant at *p* < 0.05. The Sobel test was used to calculate the significance of the indirect effect.

## Results

### Sample description

The final sample (*n* = 74) consists of 66 women (89.2%) and eight men (10.8%). The average age of the sample was 27.9 years (range: 18–46 years). The majority of study participants were unmarried (*n* = 62; 83.8%), and *n* = 24 (32.4%) were in permanent employment.

### Auditory verbal hallucinations and delusions

Delusional thoughts, as assessed by the delusions subscale of the PSYRATS, were reported by eight patients (10.8%), and AVH, as assessed by the AVH subscale of the PSYRATS, by 10 (13.5%). The mean score for the delusions subscale was M = 1.7 (SD = 5.1) in the total sample and M = 15.8 (SD = 4.8) in the affected subgroup. Means for AVH were M = 2.9 (SD = 8.0) in the total sample and M = 21.6 (SD = 8.7) in the affected subgroup.

### Childhood trauma

The CTQ showed a mean value of M = 60.4 (SD = 23.5). A total of *N* = 59 participants completed the CTQ. The frequency of the different forms of childhood abuse is specified in **Table 1**.

**Table 1:** Frequency of different forms of childhood abuse assessed with the CTQ.

	*N*	M	SD	None or minimal			Low to moderate			Moderate to severe		Severe to extreme	
				*n*		%	*n*	%		*n*	%	*n*	%
Emotional abuse	70	16.0	6.0	10	14.3		7	10.0		15	21.4	38	54.3
Physical abuse	68	9.7	5.6	37	54.4		3	4.4		9	13.2	19	27.9
Sexual abuse	64	8.8	5.6	29	45.3		5	7.8		21	32.8	9	14.1
Emotional neglect	72	16.1	5.8	10	13.9		19	26.4		14	19.4	29	40.3
Physical neglect	74	10.0	4.5	25	33.8		15	20.3		16	21.6	18	24.3

### Dissociative symptoms

The mean score of the DES was M = 23.9 (*n* = 71). The mean scores of the different subscales of the DES are shown in **Table 2**.

**Table 2:** Means of DES.

	DES-Total	Amnesia	Absorption	Depersonalization
*N*	71	73	72	72
M	23.9	12.9	32.3	18.8
SEM	17.4	15.7	19.7	21.1

### Associations between childhood trauma, dissociative symptoms, AVH, and delusions

To assess associations between childhood trauma, dissociative symptoms, AVH, and delusions, we calculated Pearson’s correlations between the different types of childhood trauma, the PSYRATS Delusional Thoughts Scale, the PSYRATS-AVH Scale, and the total score of the DES, as well as its subscales (see **Table 3**).

**Table 3:** Pearson’s correlations for CTQ-Total and its subscales and PSYRATS-AVH Scale, PSYRATS-Delusions Scale, and DES-Total and its subscales.

	PSYRATS-AVH		PSYRATS-Delusion	Absorption	Depersonalization	Amnesia	DES-Total
Emotional abuse							
*r*	0.191	− 0.022		0.365^**^	0.291^*^	0.346^**^	0.398^**^
*p*	0.113	0.857		0.002	0.016	0.004	0.001
*N*	70	70		68	68	69	67
Physical abuse							
*r*	0.081	− 0.097		0.252^*^	0.191	0.310^*^	0.258^*^
*p*	0.511	0.430		0.041	0.121	0.011	0.036
*N*	68	68		66	67	67	66
Sexual abuse							
*r*	0.453^**^	− 0.084		0.402^**^	0.408^**^	0.393^**^	0.431^**^
*p*	< 0.001	0.509		0.001	0.001	0.001	< 0.001
*N*	64	64		63	63	64	62
Emotional neglect							
*r*	0.127	0.072		0.352^**^	0.308^**^	0.328^**^	0.388^**^
*p*	0.288	0.549		0.003	0.009	0.005	0.001
*N*	72	72		70	70	71	69
Physical neglect							
*r*	0.064	− 0.012		0.300^*^	0.260^*^	0.253^*^	0.311^**^
*p*	0.587	0.919		0.010	0.027	0.031	0.008
*N*	74	74		72	72	73	71
CTQ-Total							
*r*	0.229	− 0.041		0.458^**^	0.364^**^	0.436^**^	0.464^**^
*p*	0.081	0.757		< 0.001	0.005	0.001	< 0.001
*N*	59	59		58	59	59	58

^*^ *p* < 0.05; ^**^ *p* < 0.01—levels of significance; *r*, Pearson’s correlation coefficient.

Regarding the PSYRATS scales, there was only one significant correlation. PSYRATS-AVH was positively associated with the CTQ scale sexual abuse (*r* = 0.453; *p* < 0.001).

Between the DES scores and the CTQ scores, nearly all correlations were significant, with the only exception being the lack of a significant correlation between physical abuse and depersonalization (see **Table 3**).

For the correlations between the PSYRATS scales and the DES scales, we found significant positive correlations between dissociations and hallucinations, ranging from *r* = 0.485 and 0.569 (all *p* < 0.001) for the DES subscales, and *r* = 0.544 (*p* < 0.001) for the DES-Total Scale (see also **Table 4**). The PSYRATS-Delusions Scale, on the other hand, showed weaker and, in some cases, nonsignificant associations with the DES-Total Scale (*r* = 0.259; *p* = 0.029) and its subscales (*r* = 0.218; *p* = 0.015 to *r* = 0.286; *p* = 0.065).

**Table 4:** Pearson’s correlations for the PSYRATS-AVH Scale, PSYRATS-Delusions Scale, and DES-Total and its subscales.

	PSYRATS-AVH			PSYRATS-Delusions		
	*r*	*p*	*N*	*r*	*p*	*N*
Absorption	0.485^**^	< 0.001	72	0.218	0.065	72
Depersonalization	0.524^**^	< 0.001	72	0.286^*^	0.015	72
Amnesia	0.569^**^	< 0.001	73	0.255^*^	0.029	73
DES-Total	0.544^**^	< 0.001	71	0.259^*^	0.029	71

^*^ *p* < 0.05; ^**^ *p* < 0.01—levels of significance; *r*, Pearson’s correlation coefficient.

### Mediation analyses

**Figure 1:**
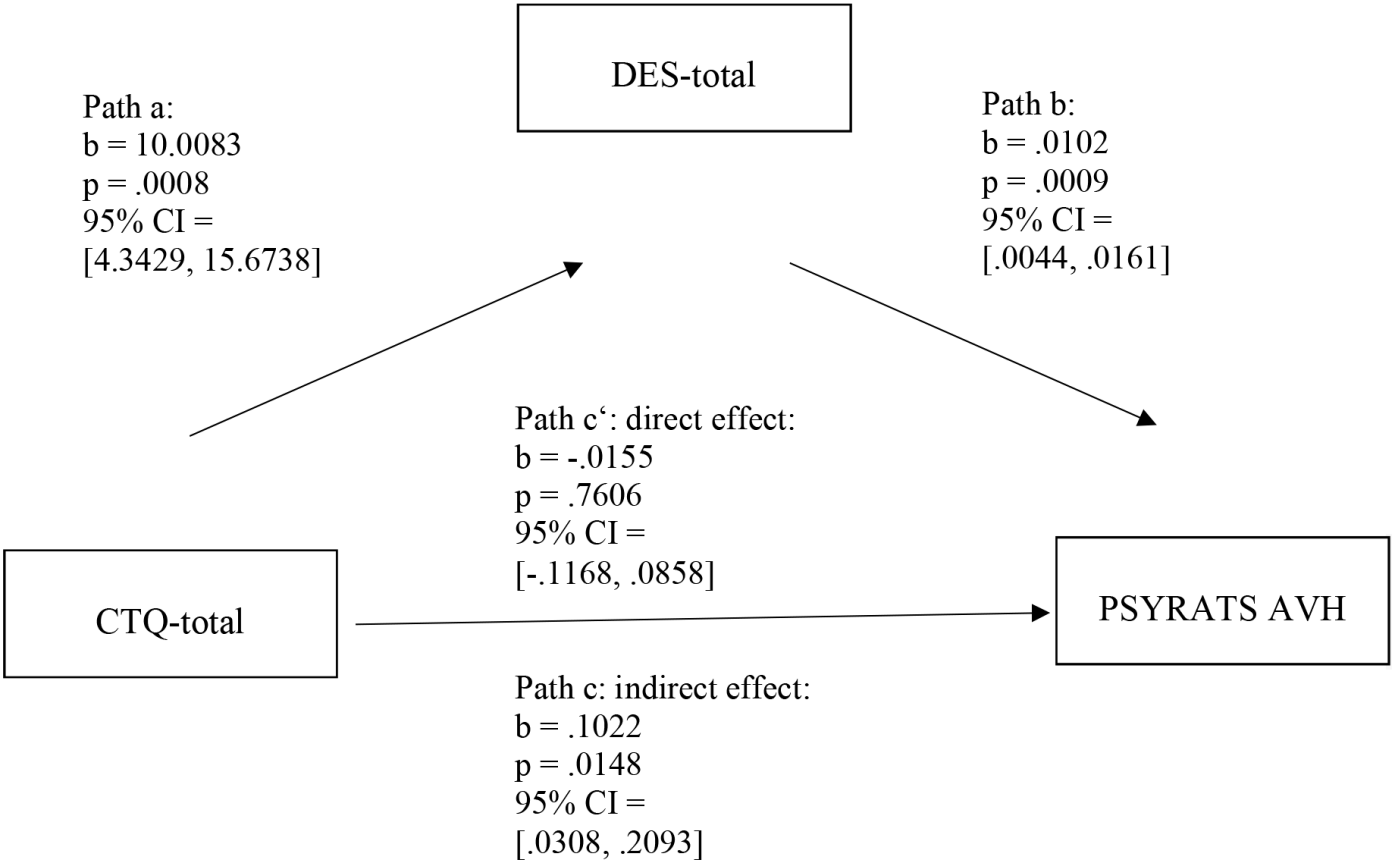
Simple mediation model with AVH as the dependent variable. *N* = 58. DES, Dissociative Experiences Scale; CTQ-Total, Childhood Trauma Questionnaire Total Score; AVH, PSYRATS-AVH Score; CI, confidence interval. The data are specified as nonstandardized B coefficients (b) and are based on 10,000 bootstrapped iterations.

To test whether dissociative symptoms, as measured with the DES, have a mediating effect between childhood maltreatment assessed with the CTQ and AVH or delusional thoughts, we conducted a mediation analysis. Our analysis showed that the DES total score significantly mediated the relationship between the CTQ total score and the PSYRATS-AVH Scale. As **Figure 1** illustrates, the unstandardized regression coefficient between the CTQ total score and the DES total score was statistically significant (path a: *b* = 10.0083; *p* = 0.0008; 95% CI: 4.3429, 15.6738), as was the unstandardized regression coefficient between the DES total score and the PSYRATS-AVH Scale (path b: *b* = 0.0102; *p* = 0.0009; 95% CI: 0.0044, 0.0161); see **Figure 1**).

**Figure 2:**
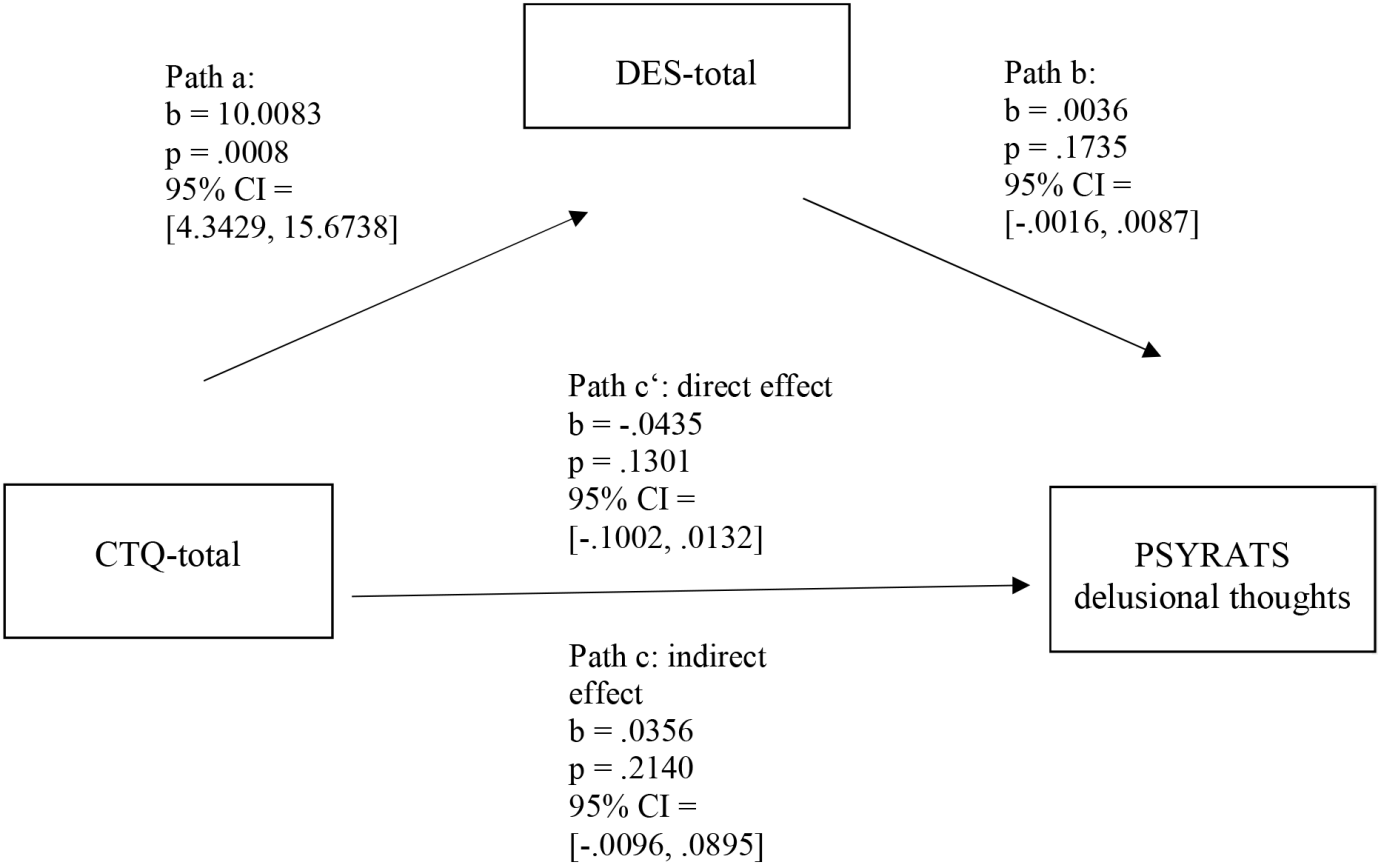
Simple mediation model with delusions as the dependent variable. *N* = 58. DES-Total, Dissociative Experiences Scale Total Score; CTQ-total, Childhood Trauma Questionnaire Total Score; PSYRATS Delusional Thoughts, PSYRATS Delusional Thoughts Score; CI, confidence interval. The data are specified as nonstandardized B coefficients (b) and are based on 10,000 bootstrapped iterations.

The DES total score did not significantly mediate the relationship between the CTQ total score and the PSYRATS-Delusions Scale. As **Figure 2** illustrates, the unstandardized regression coefficient between the CTQ total score and the DES total score was statistically significant (path a: *b* = 10.0003; *p* = 0.0008; 95% CI: 4.3429, 15.6738), but the unstandardized regression coefficient between the DES total score and the PSYRATS Delusional Thoughts Scale was not (path b: *b* = 0.0035; *p* = 0.1735; 95% CI: −0.0016, 0.0087).

## Discussion

The prevalence of delusional thoughts (10.8%) and AVH (13.5%) in our sample was lower than in previous studies of psychotic experiences in patients with BPS. Data on the frequency varied between 20% and 50% in previous studies (6). This could be explained by the recruiting procedures. Patients were recruited from a specialized ward with a standardized therapeutic approach, for which a certain level of global functioning is required. The dissociative symptomatology in our sample was, on average, of moderate severity, which is comparable to other data on the severity of dissociative symptoms in patients with BPD. For example, Zanarini et al. (53) reported that 42.0% of patients with BPD had dissociation levels of moderate severity. A total of 26.2% of patients were affected by severe dissociative symptoms, and 31.7% reported mild dissociative symptoms. The severity of childhood trauma reported by our patients (M = 60.4, SD = 23.5) is comparable to another German study (M = 63.0, SD = 21.0) by Wingenfeld and colleagues (54) and markedly higher than in a representative sample from the German population, where a CTQ total score of 35.97 is computable from the reported data (55). However, higher CTQ scores have also been reported in the literature; e.g., Kratzer et al. (56) found a mean CTQ of 78.8 in their population of BPD patients.

The relationship between childhood maltreatment and AVH, as assessed by the PSYRATS, was significantly mediated by dissociative symptoms, as measured with the DES. Dissociative symptoms, in contrast, did not significantly mediate the relationship between childhood maltreatment or violence and delusions assessed with the PSYRATS. This is in line with findings in schizophrenia, where dissociative symptoms mediated the relationship between childhood trauma and hallucinations or hallucination-proneness (40, 57, 58), but not between childhood trauma and delusions (40). These associations, however, were not replicated in a sample of first-episode psychosis patients. Contrary to previous findings, neither a significant relationship between dissociation and hallucinations nor a mediating effect of dissociation on the association between childhood trauma and hallucinations was found. Instead, in this study, dissociation mediated the relationship between childhood trauma and delusions (59). In healthy individuals, dissociation mediated the relationship between early maltreatment and both hallucination-proneness and delusional ideation (60).

Summing up our results and these studies, this might be another indication that the associations between psychotic symptoms and dissociation are not disorder-specific, but typical for early-traumatized individuals with hallucinations, delusions, and dissociative symptoms. This is another aspect that questions the strict differentiation between AVH and delusions in schizophrenia spectrum disorders on the one hand, and in borderline personality disorder on the other hand.

The current paper is strictly focused on the psychopathological symptom level, i.e., on dissociation, AVH, and delusions. These symptoms are not specific to a diagnosis and can occur in patients with different mental health problems. In particular, they can occur in borderline personality disorder and in patients with schizophrenia spectrum disorders. This is an important point, because at the disorder-specific level, an intensive discussion is still ongoing. For example, there is an ongoing discussion about dissociative symptoms in schizophrenia spectrum disorders (61), raising the question of whether dissociation could also be considered a symptom of comorbid dissociative disorder or borderline personality disorder, rather than an additional symptom in schizophrenia spectrum disorders, increased through adverse childhood events. In addition, there is evidence for a relevant level of comorbid dissociative disorders in patients with BPD (62). This raises the question of whether examining BPD in this population is, in fact, examining comorbidity with dissociative disorders. Furthermore, Sar et al. showed a considerable descriptive overlap between dissociative disorders and BPD (62–66). Following this line of thought, Meares (67) discussed the possibility that BPD itself might be a dissociative disorder. There are efforts to move beyond these discussions by referring to the Research Domain Criteria (RDoC) and the Hierarchical Taxonomy of Psychopathology [HiTOP; (68–70)]. Our work is in line with these concepts and remains at the symptom level. Based on the literature, it is assumed that there may be common pathways (57, 71, 72) leading from predisposing factors (in our case, adverse childhood events) to symptoms such as dissociation, AVH, and delusions. Describing this situation in borderline personality disorder was the objective of the current paper. These pathways could be the same for different diagnosis groups in which the symptoms occur, e.g., borderline personality disorder and schizophrenia spectrum disorders. However, different pathways might be responsible for disturbed perception in AVH and content-related thought disorder with impaired reality testing in delusions, which are, e.g., not typically seen in the context of dissociative disorders. This might explain why there was a significant mediating effect of dissociation on AVH, but not on delusions.

While the strengths of our study include the use of structured interviews for the assessment of hallucinations, delusions, and the verification of BPD, some limitations should be considered. Although only a few exclusion criteria were applied, an important limitation may relate to the representativeness of our sample. As all participants were inpatients on a specialized therapy unit, prevalence rates may not be generalizable to other BPD populations. Moreover, many patients (*n* = 30, 40.5%) received antipsychotic medication, which may also explain the lower frequency of psychotic symptoms in our sample. Both aspects might limit our conclusions. In addition, the PSYRATS used in the current study have, until now, only been validated for research in schizophrenia spectrum disorders and in affective disorders with delusions (46). However, it has been repeatedly used in studies with other patient populations, particularly borderline personality disorder (25, 73). Moreover, while we established structured diagnostics for BPD and for the absence of schizophrenia spectrum disorders, we did not specifically assess a potential comorbidity with dissociative disorders.

In many aspects, the phenomenology of the AVH in BPD is comparable to AVH in schizophrenia (8, 9, 11–14, 74), and our results add another facet similar to symptoms in schizophrenia. These symptoms should, therefore, receive the same level of attention and should not be trivialized. Trivialization may occur through the use of terms such as pseudo-hallucinations or transient paranoid ideas—terms which should no longer be used (8, 17, 75, 76). Continued use of such terms may also invalidate the patient’s experience of what are often frightening symptoms.

In contrast, hallucinations were associated with childhood trauma through mediation by dissociation and are, therefore, rather a sign of a high burden. This should raise awareness of a positive history of trauma. Treatment of traumatized patients should be trauma-sensitive, which involves siding with patients and taking them and their experiences seriously. The supportive stance should extend to their symptoms as well and should not stop when it comes to acknowledging them. For example, symptoms such as AVH should not be dismissed as pseudo-hallucinations, nor should AVH or delusions be ignored by failing to inquire about them. In schizophrenia-spectrum disorders, trauma-focused psychotherapy has been shown to reduce not only Post-traumatic stress disorder (PTSD) symptoms but also positive symptoms such as hallucinations or delusions (77). Perhaps this also applies to psychotic symptoms in BPD, and therefore innovative therapeutic approaches that are tailored to traumatized BPD patients [e.g., DBT-PTSD; (78)] might represent a promising path. This would align with the theoretical considerations of Morrison et al. (79), who proposed that both posttraumatic stress disorder and psychotic disorders fall within a spectrum of responses to traumatic life events. This spectrum is not always clearly delineated and is characterized by overlapping symptoms (intrusions, flashback experiences, hallucinations, delusions). Further research should address trauma-associated symptoms and evaluate the benefits of therapeutic approaches for each of the symptom clusters.

## Data Availability

The raw data supporting the conclusions of this article will be made available by the authors, without undue reservation.
